# Timing and localization of myasthenia gravis‐related gene expression

**DOI:** 10.1111/ejn.15382

**Published:** 2021-07-20

**Authors:** Dana L. E. Vergoossen, Arlin Keo, Ahmed Mahfouz, Maartje G. Huijbers

**Affiliations:** ^1^ Department of Human Genetics Leiden University Medical Center Leiden The Netherlands; ^2^ Leiden Computational Biology Center Leiden University Medical Center Leiden The Netherlands; ^3^ Delft Bioinformatics Lab Delft University of Technology Delft The Netherlands; ^4^ Department of Neurology Leiden University Medical Center Leiden The Netherlands

**Keywords:** brain, Dok7, Lrp4, MuSK, myasthenia gravis, neuromuscular junction

## Abstract

Myasthenia gravis (MG) is an acquired autoimmune disorder caused by autoantibodies binding acetylcholine receptors (AChR), muscle‐specific kinase (MuSK), agrin or low‐density lipoprotein receptor‐related protein 4 (Lrp4). These autoantibodies inhibit neuromuscular transmission by blocking the function of these proteins and thereby cause fluctuating skeletal muscle weakness. Several reports suggest that these autoantibodies might also affect the central nervous system (CNS) in MG patients. A comprehensive overview of the timing and localization of the expression of MG‐related antigens in other organs is currently lacking. To investigate the spatio‐temporal expression of MG‐related genes outside skeletal muscle, we used *in silico* tools to assess public expression databases. Acetylcholine esterase, nicotinic AChR α1 subunit, agrin, collagen Q, downstream of kinase‐7, Lrp4, MuSK and rapsyn were included as MG‐related genes because of their well‐known involvement in either congenital or autoimmune MG. We investigated expression of MG‐related genes in (1) all human tissues using GTEx data, (2) specific brain regions, (3) neurodevelopmental stages, and (4) cell types using datasets from the Allen Institute for Brain Sciences. MG‐related genes show heterogenous spatio‐temporal expression patterns in the human body as well as in the CNS. For each of these genes, several (new) tissues, brain areas and cortical cell types with (relatively) high expression were identified suggesting a potential role for these genes outside skeletal muscle. The possible presence of MG‐related antigens outside skeletal muscle suggests that autoimmune MG, congenital MG or treatments targeting the same proteins may affect MG‐related protein function in other organs.

## Graphical abstract

Many of the proteins involved in organizing and maintaining neuromuscular signalling cause (congenital) myasthenia gravis (MG) when their function is impaired. However, little is known on the presence and function of these proteins outside skeletal muscle. We generated a comprehensive overview of spatio‐temporal MG‐related gene expression from public human‐based databases, with a focus on the brain. These data provide a guide on tissues at risk for non‐motor symptoms in MG patients and upon MG‐related genes targeted therapy.

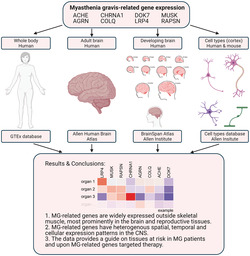

**Abbreviations**
ACh: acetylcholine
AChR: acetylcholine receptor
AHBA: Allen Human Brain Atlas
CMS: congenital myasthenic syndrome
CNS: central nervous system
CPM: counts per million reads mapped
CT: corticothalamic
CTX: isocortex
ENTI/ENT: entorhinal area
ET: extratelencephalic
GTEx: genotype‐tissue expression consortium
IT: intratelencephalic
MG: myasthenia gravis
Micro‐PVM: microglia—perivascular macrophages
MuSK: muscle‐specific kinase
NMJ: neuromuscular junction
NP: near projecting
OPC: oligodendrocyte progenitor cells
PT: pyramidal tract
SMC‐Peri: smooth muscle cell ‐ pericyte
snRNA‐seq: single nucleus RNA sequencing
TPE: temporal, perirhinal and ectorhinal
TPM: transcripts per million
VLMC: vascular leptomeningeal cells

## 1 INTRODUCTION

Myasthenia gravis (MG) is an antibody‐mediated autoimmune disorder hallmarked by fatigable skeletal muscle weakness. This muscle weakness results from autoantibodies targeting essential proteins at the neuromuscular junction (NMJ). Till date, four antigens have been described: acetylcholine receptors (AChR), muscle‐specific kinase (MuSK), low‐density lipoprotein receptor‐related protein 4 (Lrp4) and agrin (Gilhus & Verschuuren, 2015). These proteins converge on a single pathway essential for establishing and maintaining NMJs and facilitating neuromuscular transmission (Burden et al., 2018). Consequently, binding of autoantibodies interferes with the function of these proteins, resulting in failure of neuromuscular transmission and subsequent muscle fatigue and paralysis.

Even though most knowledge on these proteins relates to their function in the NMJ, they are also expressed in tissues other than the skeletal muscle, like the retina, lung and brain (Bezakova & Ruegg, 2003; Burden et al., 2018; Carlisle et al., 2004; Tian et al., 2006). Insight in the localization of expression of these genes/proteins and their roles in other organs is important because: (1) Other tissues may also be affected by the autoantibodies in MG patients, and (2) new therapeutic strategies targeting these MG‐related genes/proteins are emerging; therefore, knowledge on their localization may identify potential off‐target effects. A comprehensive overview of MG‐related gene expression in different human tissues however is lacking.

Evidence of other organs being affected by MG autoantibodies is mostly focused on the central nervous system (CNS). The non‐motor symptoms include pain, cognitive dysfunction, fatigue and sleep disturbances (Bhagavati et al., 2007; Mao et al., 2015; Paul et al., 2000; Ruiter et al., 2020; Tong et al., 2018). Although AChR MG serum antibodies were reported not to bind neuronal AChR (Whiting et al., 1987) and immunostaining with MuSK autoantibodies seems challenging on brain sections (personal observation), passive transfer of patient‐derived MuSK and AChR antibodies resulted in behavioural deficits and EEG abnormalities in mice (Fulpius et al., 1977; Sabre et al., 2019). Moreover, AChR autoantibodies have been detected in cerebrospinal fluid (Keesey et al., 1978; Lefvert & Pirskanen, 1977; Müller et al., 1987). Mutations in NMJ genes may furthermore result in congenital myasthenic syndrome (CMS) and sometimes give CNS abnormalities (Engel, 2018). Although CNS defects are not at the foreground of clinical symptoms in MG patients, these observations suggest that autoantibodies may have detrimental effects in the CNS when they are able to cross the blood–brain barrier.

If indeed MG‐related autoantibodies are able to affect their antigens in other organs several important questions arise, for example, (1) do these proteins, like in the NMJ, converge on a similar pathway in these organs and (2) which cells are responsible for this expression. We therefore investigated the spatio‐temporal expression patterns of MG‐related genes in healthy human tissues, with a focus on the CNS, using a range of publicly‐available expression databases. We selected the four NMJ genes which encode known antigens for autoantibodies in MG (*AGRN, CHRNA1, LRP4, MUSK*) and four other NMJ genes involved in maintaining NMJ neurotransmission and where mutations can cause CMS (acetylcholine esterase (*ACHE*)*,* collagen Q (*COLQ*)*,* downstream of kinase‐7 (*DOK7*)*,* and rapsyn (*RAPSN*)) (Table 1) (Engel, 2018). We will refer to these eight genes and their gene products as MG‐related genes.

**TABLE 1:** The MG‐related genes, their function at the NMJ and association with autoimmune MG or CMS (Burden et al., 2018; Engel, 2018; Gilhus & Verschuuren, 2015; Zisimopoulou et al., 2014)

Gene symbol	Encoded protein	Function at NMJ	Role in CMS or autoimmune MG
*ACHE*	Acetylcholine esterase	Breaks down the neurotransmitter acetylcholine (ACh), thereby halting neuromuscular transmission and muscle contraction.	12.53% of CMS patients have a AChE deficiency. AChE is the main target for symptomatic treatment of autoimmune MG patients.
*AGRN*	Agrin	Trophic signalling molecule released by the presynaptic motor nerve terminal to induce and maintain postsynaptic differentiation. Agrin lingers in the basal lamina, can bind Lrp4 and thereby activates MuSK and AChR clustering.	Mutations in *AGRN* are a rare cause of CMS (0.58%). Autoantibodies to agrin are thought to cause autoimmune MG in a small subset of patients.
*CHRNA1*	Nicotinic acetylcholine receptor α subunit	Muscle‐specific subunit of the AChR required for neuromuscular transmission and location for ACh binding. Upon binding of ACh to the AChR, its ion channel opens which depolarizes the muscle end plate, and may induce an action potential and muscle contraction.	~50% of CMS patients have an AChR deficiency caused by mutations in one of the AChR subunits leading to kinetic defects and myasthenic symptoms. ~80% of autoimmune MG patients have autoantibodies to the AChR. The α subunit contains the main immunogenic region.
*COLQ*	Collagen Q	Collagenic subunit anchoring AChE to the basal lamina and thereby responsible for ACh breakdown. Also interacts with MuSK.	The pathomechanism of AChE and MuSK CMS as well as autoimmune MG are known to affect ColQ function.
*DOK7*	Downstream of kinase 7	Cytoplasmic adaptor of MuSK required for regulating the kinase activity of MuSK, subsequent AChR clustering and NMJ formation.	9.75% of CMS patients have a mutation in *DOK7*. *DOK7* gene therapy rescued the phenotype of a Dok7 CMS mouse model.
*LRP4*	Low‐density lipoprotein receptor‐related protein 4	The agrin receptor that directly interacts with MuSK and further activates AChR clustering and postsynaptic differentiation. Lrp4 is also critical for presynaptic differentiation.	0.56% of CMS patients have mutations in *LRP4*. 1–2% of MG patients have Lrp4 autoantibodies. MuSK autoantibodies inhibit MuSK‐Lrp4 interaction, obstructing normal trophic signalling at the NMJ and induce myasthenia.
*MUSK*	Muscle‐specific kinase	Orchestrates anterograde trophic signalling at the NMJ, resulting in AChR clustering. It is also required for retrograde signalling towards presynaptic differentiation of the motor neuron end plate.	0.28% of CMS patients have a mutation in *MUSK*. Autoantibodies to MuSK cause autoimmune MG in 5–8% of patients.
*RAPSN*	Rapsyn	Cytoplasmic anchor that facilitates AChR clustering.	14.21% of CMS patients have a rapsyn deficiency.

## 2 MATERIALS AND METHODS

### 2.1 Genotype‐tissue expression consortium (GTEx)

The expression of MG‐related genes was analysed across 54 nondiseased human tissues using RNA sequencing data of the nearly 1,000 individuals from the open access genotype‐tissue expression consortium (GTEx) version 8. For *ACHE* (ENSG00000087085) and *AGRN* (ENSG00000188157), all isoforms were annotated using the Ensembl database. The median gene‐level transcripts per million (TPM) for all MG‐related genes were downloaded per tissue. Because it is known that *ACHE* and *AGRN* produce different splice variants, of which only one is relevant for NMJs, *ACHE‐207* (ENST00000428317.5) and *AGRN‐208* (ENST00000620552.4) isoform data were downloaded separately. The anterior cingulate cortex, frontal cortex and cerebellar hemisphere were removed because they were covered in the cortex or cerebellum respectively. EBV‐transformed lymphocytes and cultured fibroblasts were removed, because they do not naturally occur in healthy humans. Tissues (rows) and genes (columns) were hierarchically clustered using hclust function in R with Eucledian distance and average linkage. Qualitative expression levels are used as follows: low (Log10 < 1), moderate (Log10 1–1.5), high (Log10 > 1.5).

### 2.2 Allen Human Brain Atlas

The Allen Human Brain Atlas (AHBA) includes anatomically mapped Oligo‐dT primed microarray data of 3,702 samples from six neurotypical individuals (5 males and 1 female, mean age 42, range 24–57 years) (Hawrylycz et al., 2012). The expression of the MG‐related genes was mapped across regions of the human brain using BrainScope (Huisman et al., 2017). The MG‐related gene‐probes used are marked in Table S1. All probes were confirmed to align to their respective MG‐related gene using the Ensembl database. *AGRN* probe A_24_P358462 (#) detected the NMJ‐specific *AGRN‐208* isoform, able to induce AChR clustering and clusters separately from the probe used in the other analyses (*) (Figure S3a) (McMahan et al., 1992). For *ACHE*, the available probe did not selectively bind to *ACHE‐207*. The expression of MG‐related genes was downloaded from http://human.brain-map.org/. Log2‐transformed expression values were converted into z‐scores normalized per donor. The median z‐score of each gene was taken across the set of 22 nonoverlapping brain regions. Finally, the median z‐score of every MG gene across the six donors was taken. Hierarchical clustering was done as described above. The nature of these data only allowed comparison of z‐scores across anatomical brain areas within one gene, which is why the term relative expression is used. Qualitative expression levels are used as follows: low (Z < 0), moderate (Z 0–1), high (Z > 1).

### 2.3 BrainSpan atlas of the developing human brain

The BrainSpan atlas of the developing human brain includes 42 healthy human brains, ranging in age from 8 weeks postconception to 40 years old, from which a total of 524 anatomically annotated samples were taken (Miller et al., 2014). Gene expression was determined using RNA sequencing and visualized using BrainScope (Huisman et al., 2017). *ACHE‐207* and *AGRN‐208* isoforms could not be distinguished in this dataset. MG‐related genes *CHRNA1*, *DOK7*, *MUSK* and *RAPSN* were not present in BrainScope, since genes with RPKM‐value above 1 in less than 20% of all samples were removed. For those genes, we used the brain‐map.org portal to plot their expression (Figure S4).

### 2.4 Single‐cell analyses

Single‐nucleus RNA sequencing (snRNA‐seq) data from multiple human cortical regions and single‐cell RNA sequencing data from the whole mouse cortex were downloaded from the Allen Institute (https://portal.brain-map.org/atlases-and-data/rnaseq). For the human dataset, the donors included 4 males and 4 females (age 24–66) without a history of neuropsychiatric or neurological conditions (Hodge et al., 2019). From 4 donors, multiple cortical areas were sampled postmortem and from 4 donors the medial temporal gyrus was removed during neurosurgery. For the mouse dataset, 538 animals were used from multiple transgenic lines to enrich for rare cell types, all on a C57BL/6 J background (Yao et al., 2021).

Sample processing and analysis methods have been described previously (Hodge et al., 2019; Yao et al., 2021). Briefly, the SMART‐seq method yielded transcriptome profiles for 10,708 glutamatergic neurons, 4,297 GABAergic neurons and 923 nonneuronal cells for the human dataset and 40,276 glutamatergic neurons, 22,573 GABAergic neurons and 1,958 nonneuronal cells for the mouse dataset. The trimmed‐mean gene expression (counts per million reads mapped [CPM]) data per cluster was downloaded for 8 MG‐related genes and grouped in the cell type subclasses defined in the sampling strategy. In the mouse dataset, we selected the analogous cell type subclasses that are present in the human dataset (Hodge et al., 2019).

## 3 RESULTS

### 3.1 Expression of MG‐related genes in muscle and brain regions

**FIGURE 1:**
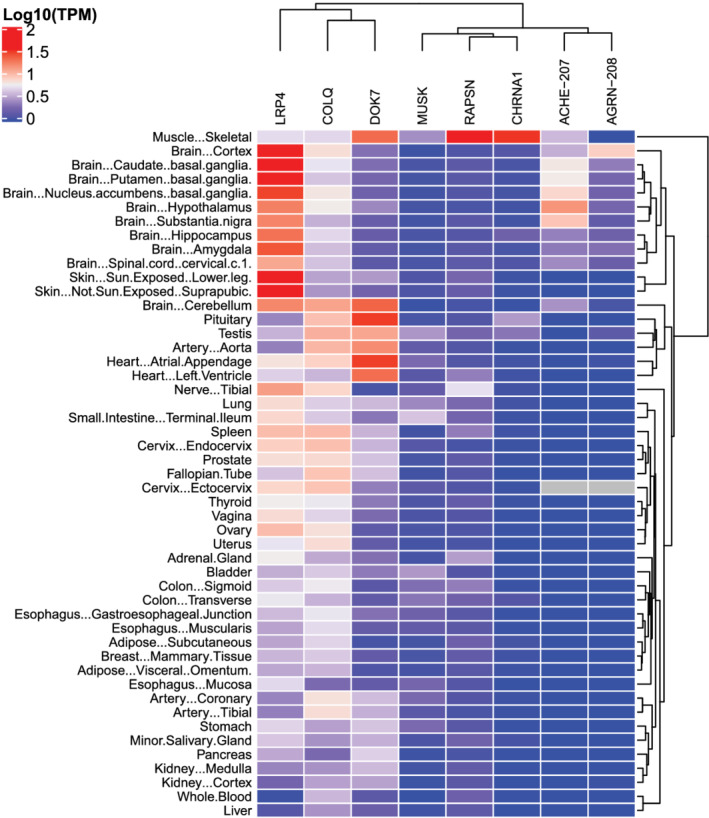
Overview of tissue‐specific expression of MG‐related genes reveals most co‐expression in skeletal muscle, the CNS and parts of the male and female reproductive system. The heatmap shows the median expression of each MG‐related gene (column) in TPM across human tissues (rows) using data from GTEx V8. Average expression is reported as Log10(TPM + 1)

Expression of MG‐related genes was assessed in human tissues using GTEx data. For *ACHE* and *AGRN*, specific splice variants (*ACHE‐207* and *AGRN‐208*) are known to be (NMJ) synapse‐specific (McMahan et al., 1992; Zimmermann, 2013). *ACHE‐207* and *AGRN‐208* were indeed specifically expressed in skeletal muscle and/or brain regions, in contrast to other isoforms (Figure S1). Because of their relevance for the NMJ and association with autoimmune MG, *ACHE‐207* and *AGRN‐208* were selected for further analysis (Figure 1). For completeness, the analysis was also performed using gene counts (i.e., including all isoforms) in Figure S2.

Hierarchical clustering revealed that skeletal muscle segregates from all other tissues based on expression of MG‐related genes, supporting the unique combined role of these genes in the NMJ (Figure 1). Agrin is secreted by motor neurons, explaining the absence of *AGRN‐208* expression in skeletal muscle. The absence of *AGRN‐208* in the tibial nerve may be explained by localization of mRNA to subcellular compartments in long axons (Jung et al., 2012). Such transcripts are missed when sampling the nerve nucleus.

Cerebral brain areas clustered together close to skeletal muscle with expression of nearly all MG‐related genes, confirming expression of these genes in healthy adult human brain. All MG‐related genes could be detected in areas such as hippocampus and basal ganglia, although the level of expression differed compared to skeletal muscle. The cerebellum clustered separately from the cerebrum with moderate expression of *DOK7*, *LRP4* and *COLQ*. Transcriptomic separation of cerebellum and cerebrum is observed for many other genes (Huisman et al., 2017). Outside the brain, almost all MG‐related genes are found in the testis and the ectocervix. Other components of the male and female reproductive systems cluster together with moderate expression of *LRP4* and *COLQ* and low expression of *DOK7*, *MUSK* and/or *RAPSN*. Varying expression for subsets of MG‐related genes could be detected in the remaining tissues.

We observed three clusters of the MG‐related genes (Figure 1). *LRP4*, *COLQ* and *DOK7* have relatively ubiquitous expression across all tissues. Notably, *LRP4* was most expressed in the brain and the skin, *COLQ* in the cerebellum, pituitary gland, testis and heart, and *DOK7* in the heart and pituitary gland. In contrast, *MUSK*, *RAPSN* and *CHRNA1* showed low expression in a more limited subset of tissues. *MUSK* expression is highest in the small intestine, bladder and testis. *RAPSN* and *CHRNA1* expression was largely restricted to skeletal muscle, with some additional expression in the tibial nerve, and testis and pituitary gland respectively. Finally, the *ACHE‐207* and *AGRN‐208* isoforms clustered together based on their expression in the brain and ectocervix. Taken together, the expression of MG‐related genes is prominent in skeletal muscle and the brain, but individual genes are also expressed in other tissues of the human body.

### 3.2 NMJ genes do not share anatomical expression patterns in the brain

**FIGURE 2:**
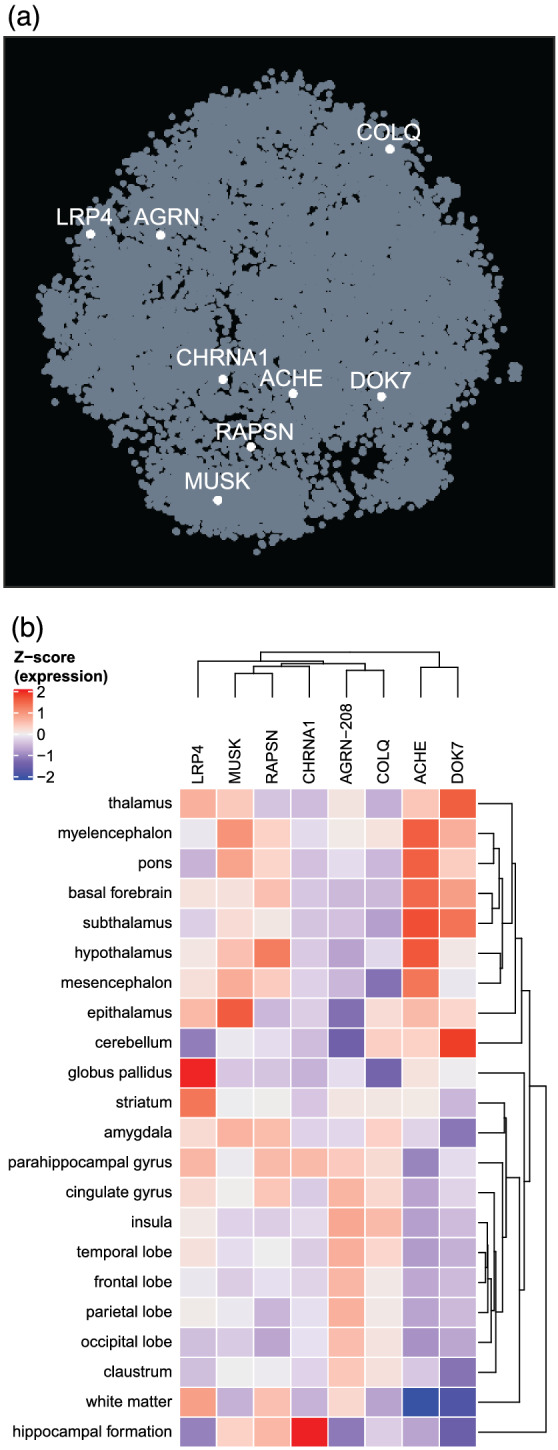
Spatial expression of MG‐related genes in the adult human brain. Co‐expression analysis of MG‐related genes, based on their distribution in a t‐SNE embedding, reveals that they do not share spatial coexpression (a). The t‐SNE from BrainScope shows an embedding of all genes (dots) based on their expression pattern across the whole brain. (b) A heatmap showing the average expression of MG genes across distinct (non‐overlapping) brain regions. Expression values represent the average z‐score normalized expression per gene (i.e., relative expression across brain regions)

Compared to the 10 brain regions in the GTEx database, the AHBA provides a high‐resolution map of relative gene expression across the adult human brain. Strong correlation between spatial expression patterns would become apparent when genes cluster in the same region of the t‐SNE plot (Figure 2a). However, MG‐related genes are scattered across the t‐SNE plot, suggesting they do not share spatial expression patterns across the brain.

For hierarchical clustering analysis of distinct brain regions, the NMJ‐specific isoform of agrin (*AGRN‐208)* was included (Figure 2b) while a heatmap including all *AGRN* isoforms can be found in Figure S3b. The hippocampus clustered separately from all other brain regions with the unique pattern of high relative expression of *CHRNA1*, *RAPSN* and *MUSK* (Figure 2b). The remaining anatomical brain regions separated in two large clusters enriched in the developmentally early and late regions respectively. The early developmental cluster with hindbrain, midbrain and diencephalon was characterized by high relative expression of *DOK7* and *ACHE* and predominant low relative expression of *COLQ* and *AGRN‐208*. In contrast, the late developmental cluster, with the basal ganglia, limbic system and cortical regions, was characterized by moderate relative expression of *COLQ* and *AGRN‐208* and low relative expression of *DOK7* and *ACHE*. Furthermore, expression is highest in a unique structure for most genes. *DOK7* in cerebellum, *CHRNA1* in hippocampus, *MUSK* in epithalamus, *LRP4* in globus pallidus and *RAPSN* in the hypothalamus. Taken together, this suggests that MG‐related genes are likely not active in similar pathways in the CNS as they do not share spatial expression patterns. However, subsets of these genes may be involved in signalling in similar structures.

### 3.3 NMJ genes do not co‐segregate in developmental time in the human brain

**FIGURE 3:**
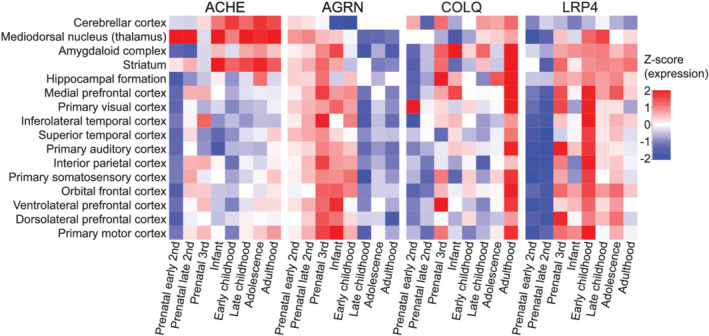
Overview of the expression patterns of four MG‐related genes throughout human brain development. *ACHE*, *AGRN*, *COLQ* and *LRP4* are expressed during different phases of human brain development

To investigate whether expression of these genes follows a temporal pattern in brain development, we used the BrainSpan atlas visualized in the BrainScope browser (Huisman et al., 2017; Miller et al., 2014). The expression of *ACHE, AGRN, COLQ* and *LRP4* did not follow the same pattern across human brain development (Figure 3). *AGRN* was particularly expressed until early childhood while *LRP4* was expressed from the 3rd prenatal stage into adulthood. *ACHE* expression was predominantly restricted to the cerebellum, thalamus, amygdala and striatum from birth until adulthood. *COLQ* is highest across the entire brain in adult life, although various brain regions also show high expression in the last prenatal stage. *CHRNA1*, *DOK7*, *MUSK* and *RAPSN* could not be studied with BrainScope, but the brain‐map.org portal confirms the low and unique expression patterns of these genes (Figure S4). In sum, spatio‐temporal expression of MG‐related genes is heterogenous in the human brain.

### 3.4 MG‐related genes do not coexpress in the same cell types in the cortex

**FIGURE 4:**
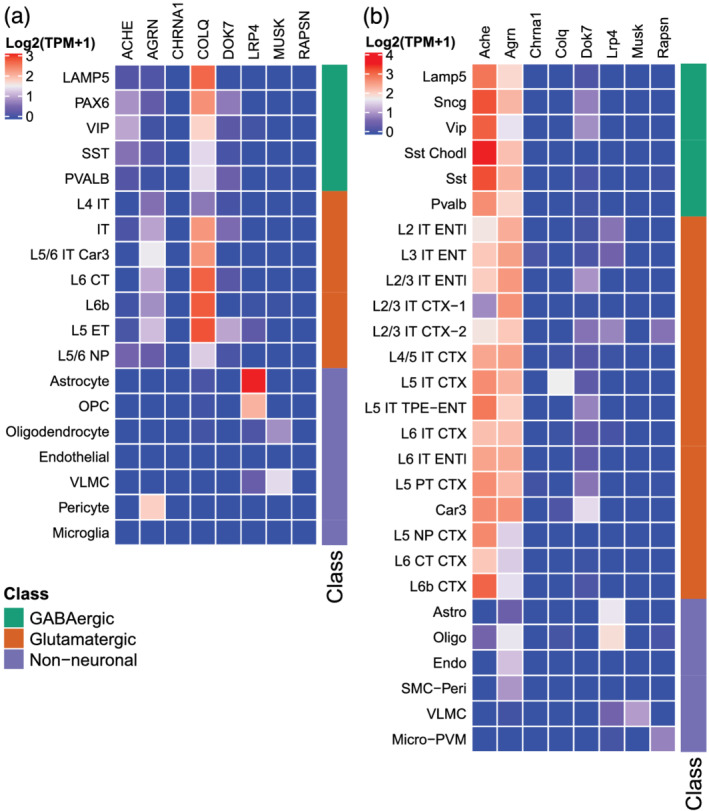
Cortical cell type expression of MG‐related genes. Heatmap of adult human cortical cell types (a). Heatmap of adult mouse cortical cell types (b). CT, corticothalamic; CTX, isocortex; ENTI/ENT, entorhinal area; ET, extratelencephalic; IT, intratelencephalic; micro‐PVM, microglia—perivascular macrophages; NP, near projecting; OPC, oligodendrocyte progenitor cells, PT, pyramidal tract; SMC‐Peri, smooth muscle cell—pericyte; TPE, temporal, perirhinal and ectorhinal; VLMC, vascular leptomeningeal cells. Average expression is reported as Log10(TPM + 1)

To understand which cell types are responsible for the regional expression of MG‐related genes in the CNS, we explored a snRNAseq dataset (Hodge et al., 2019). The expression of *CHRNA1* and *RAPSN* could not be detected in cortical cell types (Figure 4a). This is in line with the very low to absent expression in the bulk RNAseq datasets of the cortical regions (Figures 1 and S4). Interestingly, *MUSK* and *LRP4* were uniquely expressed in non‐neuronal cell types, with *MUSK* solely in vascular leptomeningeal cells (VLMC) and oligodendrocytes and *LRP4* predominantly in astrocytes and oligodendrocyte progenitor cells (Figure 4a). *ACHE*, *AGRN*, *COLQ* and *DOK7* were predominantly expressed in glutamatergic and/or GABAergic neurons; although, *AGRN* was also expressed in pericytes. Overall, a variety of cell types seem to be responsible for expression of MG‐related genes in the cortex.

As many preclinical studies related to MG are done in rodents, it is relevant to know how the expression of MG‐related genes compares between humans and rodents. In mouse cortical regions, similar cell types expressed *MUSK*, *LRP4*, *CHRNA1* and *RAPSN*, suggesting adequate translatability of results (Figure 4b). In contrast, *ACHE* and *AGRN* expression was ubiquitously high in mouse neuronal cell types, but much more restricted to GABAergic or glutamatergic cell types respectively in the human cortex. For *COLQ*, the expression is ubiquitously high in human neuronal cell types, but very much restricted to a single glutamatergic subclass in the mouse. For these genes, translatability of functional studies to humans may thus be limited.

## 4 DISCUSSION

This study confirms that many of the MG‐related genes are widely expressed outside skeletal muscle, with the exact pattern of involved organs varying per gene. Within the CNS, these genes furthermore show heterogenous spatial, temporal and cellular expression patterns. This suggests that these genes do not converge on a single pathway in the brain or other human tissues. It furthermore provides valuable insights for new research questions and hypotheses, explaining possible non‐motor symptoms in myasthenic syndromes or off‐target effects of MG‐related targeted treatments.

Validity of these expression data is supported by studies confirming the presence of these MG‐related proteins outside skeletal muscle. Congruent with the observed expression in testis, agrin, MuSK and rapsyn are present in human sperm, (Kumar et al., 2006; Kumar & Meizel, 2005). Dok7 protein was detected in heart and brain, and MuSK in bladder, heart, lung and liver (Garcia‐Osta et al., 2006; Okada et al., 2006). Agrin is present in glutamatergic neurons and increasingly surrounding brain microvasculature during development of the blood–brain barrier, validating the expression in pericytes and during prenatal development (Barber & Lieth, 1997; Cohen et al., 1997). Lrp4 is indeed abundantly present in the brain, most prominently in astrocytes (Karakatsani et al., 2017; Tian et al., 2006). Not all observations however match with previous studies. Dok7 was not detected in liver and spleen before (Garcia‐Osta et al., 2006; Okada et al., 2006) and MuSK protein was detected in spleen but is not present in our dataset. MuSK and agrin were found in glutamatergic neurons in the mouse cortex, while *MUSK* is only expressed in VLMCs and *AGRN* also in GABAergic neurons in the cell‐type analysis (Ksiazek et al., 2007). It is well established that RNA levels do not always translate to protein; however, discrepancies may also be explained by sensitivity and type of detection method, timing or location of sampling, or differences between rodents and humans. The Allen Institute databases furthermore cover a limited number of individuals, making it difficult to translate these observations to a larger heterogenous population. Cell type‐specific expression is currently only available for the cortex, covering a fraction of the cellular complexity in the CNS. More single cell data is anticipated as this field is rapidly expanding. Keeping these limitations in mind, our gene expression analysis method provides biological rationale to further investigate a possible role for candidate genes in newly identified target tissues. This is particularly important for MG‐related genes like *Musk*, *Agrin, Dok7* and *Lrp4,* for which the role in other organs may be overlooked*,* because null mice die at birth of impaired breathing due to dysfunctional NMJs (Dechiara et al., 1996; Gautam et al., 1996; Okada et al., 2006; Weatherbee et al., 2006).

Surprisingly, many of the MG‐related genes were found in parts of the male and female reproductive system. Their role there remains to be uncovered. However, fertility issues have not been described in MG patients nor in mouse models lacking these genes. Interestingly, quite a number of MG‐related genes have prominent expression in non‐neuronal cell types in the cortex, suggesting a role for these genes outside a synaptic context. Expression of *MUSK* in VLMCs has not been described before and provides an important clue for further research into the role of MuSK in the brain. Till date, for a limited number of MG‐related genes the function was studied in the CNS. Reduced agrin, Lrp4 or MuSK levels impaired cortical synaptogenesis and/or hippocampal functioning in mice (Garcia‐Osta et al., 2006; Gomez et al., 2014; Karakatsani et al., 2017; Ksiazek et al., 2007; Sun et al., 2016; Zhang et al., 2019). This fits the observation that all three genes are expressed in the hippocampus, although the prominent effects of reducing MuSK or agrin were surprising considering the relatively low expression of *MUSK* and *AGRN‐208*. Whether these three genes converge on the same pathway in the hippocampus is uncertain. Agrin was shown to induce MuSK signalling through Lrp4 in hippocampal astrocytes (Sun et al., 2016). However, the role of agrin and Lrp4 in hippocampal neurogenesis seems to be mediated by Ror2, instead of MuSK, supporting that subsets of MG‐related genes may also work together with other proteins (Zhang et al., 2019). Taken together, MG‐related genes may participate in similar pathways under certain condition in the hippocampus, but little is known about their role in the rest of the brain. The spatial, temporal and cellular resolution of our analysis can guide future studies to specific anatomical regions, developmental phase and cell types in the brain.

The expression of MG‐related genes outside skeletal muscle suggests that other organs may be at risk for impairment by autoantibodies. In autoimmune MG and CMS patients, skeletal muscle weakness is clearly at the foreground of symptoms. The presence of these genes in the CNS suggests that the reported CNS‐related symptoms in subsets of MG patients may be due to autoantibodies binding their target if they cross the blood–brain barrier, although this is not expected in patients with an intact blood–brain barrier (Bhagavati et al., 2007; Mao et al., 2015; Paul et al., 2000; Ruiter et al., 2020; Tong et al., 2018). In CMS patients, a mutational bias may occur as observed genetic defects in these genes are likely to result in a sufficiently mild phenotype to allow development. Absence of other symptoms may be explained by MG‐related gene products (1) having low levels of expression, (2) being inaccessible to autoantibodies, (3) not fulfilling an essential function, (4) having alternative splice variants or (5) being differentially posttranslationally modified masking relevant epitopes. Furthermore, observed non‐motor symptoms or comorbidities may also be due to underlying immune dysfunction, thymoma's or treatment side‐effects. Future studies are needed to investigate whether CNS‐related or other non‐motor symptoms in MG patients are due to autoantibody binding or gene dysfunction not yet recognized.

MG‐related genes or proteins are interesting targets for treatment of neuromuscular diseases through strengthening NMJs and improving or maintaining muscle function (Ohno et al., 2017). *DOK7* gene therapy, agonistic MuSK antibodies and agrin biologicals have proven beneficial in several mouse models for ALS, Dok7 CMS, Emery Dreifuss muscular dystrophy, spinal muscular atrophy or sarcopenia (Arimura et al., 2014; Boido et al., 2018; Cantor et al., 2018; Hettwer et al., 2014; Miyoshi et al., 2017; Sengupta‐Ghosh et al., 2019). Off‐target effects in other organs have not been reported, but also not specifically investigated. Our data furthermore identified some differences between mouse and human MG‐related gene expression which emphasizes where caution is needed for accurate interpretation and translatability of studies using mouse models. For further clinical development, our data provides clear guidance as to which organs may be at risk for off‐target effects. Since many of the MG‐related genes are expressed in the brain, a treatment strategy that does not cross the blood–brain barrier is recommended.

This hypothesis‐free approach to study timing and localization of MG‐related gene expression suggests wide‐spread expression of these genes outside skeletal muscle. These insights can guide future studies to uncover their role in other organs and guide pre‐clinical development of related novel therapeutics.

## CONFLICT OF INTEREST

LUMC receives royalties from IBL for a MuSK diagnostic assay. LUMC and MGH receive royalties from licensed patent applications on MuSK‐related research. The other authors report no conflict of interests.

## AUTHOR CONTRIBUTIONS

DV, AM and MH designed the study, analysed the data and wrote the paper. AK analysed the data.

### PEER REVIEW

The peer review history for this article is available at https://publons.com/publon/10.1111/ejn.15382.

## Supporting information

**Table S1.** Genes and probes used for MG‐related genes from the AHBA. * used for analysis, unless otherwise specified. # detects the AGRN‐208 isoform able to induce AChR clustering at the NMJ.
**Figure S1.** Expression of *ACHE* isoforms (A) and *AGRN* isoforms (B) extracted from the GTEx database. Expression of synapse‐specific *ACHE‐207* isoform (red) is limited to brain areas and skeletal muscle. Expression of NMJ‐specific *AGRN‐208* isoform (red) is limited to brain areas. Grey isoforms are non‐protein coding according to Ensembl (GRCh38). * isoforms identified in earlier assemblies of the human genome.
**Figure S2.** Hierarchical clustering of gene expression in the GTEx database using all isoforms of *ACHE* and *AGRN*.
**Figure S3.** Heatmaps from Allen human brain atlas. Relative expression of different agrin probes across distinct brain areas (A). # probe that selectively detects NMJ‐relevant isoform of agrin. * probe for AGRN used in the BrainScope tool detecting all AGRN isoforms. Hierarchical clustering using probe for AGRN that does not discriminate between isoforms (*) (B).
**Figure S4.** Gene expression of MG‐related genes during human development do not show the same pattern across development. *CHRNA1* expression is increased across development restricted to the hippocampus. *DOK7* shows a pattern of increasing expression across development, with highest expression in the cerebellum and thalamus. *MUSK* is lowly expressed during prenatal development in the hippocampus, amygdala and cerebellum. *RAPSN* shows very limited expression across development, restricted to some subcortical regions of the brain. Visualized using the BrainSpan portal.

## Data Availability

The data that support the findings of this study are available from GTEx version 8 and the Allen Institute as described under the relevant subheadings of the methods section. Scripts to generate all the results presented in this manuscript can be found online at: https://github.com/ahmedmahfouz/MG-analysis.

## References

[ejn15382-bib-0001] Arimura, S., Okada, T., Tezuka, T., Chiyo, T., Kasahara, Y., Yoshimura, T., Motomura, M., Yoshida, N., Beeson, D., Takeda, S., & Yamanashi, Y. (2014). DOK7 gene therapy benefits mouse models of diseases characterized by defects in the neuromuscular junction. Science, 345, 1505–1508. 10.1126/science.1250744 25237101

[ejn15382-bib-0002] Barber, A. J., & Lieth, E. (1997). Agrin accumulates in the brain microvascular basal lamina during development of the blood‐brain barrier. Developmental Dynamics, 208, 62–74. 10.1002/(SICI)1097-0177(199701)208:1<62::AID-AJA6>3.0.CO;2-# 8989521

[ejn15382-bib-0003] Bezakova, G., & Ruegg, M. A. (2003). New insights into the roles of agrin. Nature Reviews Molecular Cell Biology, 4, 295–309. 10.1038/nrm1074 12671652

[ejn15382-bib-0004] Bhagavati, S., Maccabee, P. J., & Chari, G. (2007). Is cerebral involvement an occasional feature of muscle‐specific kinase antibody‐positive syndrome? European Journal of Neurology, 14, e21–e22. 10.1111/j.1468-1331.2007.01873.x 17661994

[ejn15382-bib-0005] Boido, M., De Amicis, E., Valsecchi, V., Trevisan, M., Ala, U., Ruegg, M. A., Hettwer, S., & Vercelli, A. (2018). Increasing agrin function antagonizes muscle atrophy and motor impairment in spinal muscular atrophy. Frontiers in Cellular Neuroscience, 12, 17. 10.3389/fncel.2018.00017 29440993 PMC5797594

[ejn15382-bib-0006] Burden, S. J., Huijbers, M. G., & Remedio, L. (2018). Fundamental molecules and mechanisms for forming and maintaining neuromuscular synapses. International Journal of Molecular Sciences, 19(2), 490. 10.3390/ijms19020490 PMC5855712 29415504

[ejn15382-bib-0007] Cantor, S., Zhang, W., Delestree, N., Remedio, L., Mentis, G. Z., & Burden, S. J. (2018). Preserving neuromuscular synapses in ALS by stimulating MuSK with a therapeutic agonist antibody. eLife, 7, e34375. 10.7554/eLife.34375 29460776 PMC5837562

[ejn15382-bib-0008] Carlisle, D. L., Hopkins, T. M., Gaither‐Davis, A., Silhanek, M. J., Luketich, J. D., Christie, N. A., & Siegfried, J. M. (2004). Nicotine signals through muscle‐type and neuronal nicotinic acetylcholine receptors in both human bronchial epithelial cells and airway fibroblasts. Respiratory Research, 5, 27. 10.1186/1465-9921-5-27 15588326 PMC544394

[ejn15382-bib-0009] Cohen, N. A., Kaufmann, W. E., Worley, P. F., & Rupp, F. (1997). Expression of agrin in the developing and adult rat brain. Neuroscience, 76, 581–596. 10.1016/S0306-4522(96)00345-4 9015340

[ejn15382-bib-0010] Dechiara, T. M., Bowen, D. C., Valenzuela, D. M., Simmons, M. V., Poueymirou, W. T., Thomas, S., Kinetz, E., Compton, D. L., Rojas, E., Park, J. S., Smith, C., DiStefano, P. S., Glass, D. J., Burden, S. J., & Yancopoulos, G. D. (1996). The receptor tyrosine kinase MuSK is required for neuromuscular junction formation in vivo. Cell, 85, 501–512. 10.1016/S0092-8674(00)81251-9 8653786

[ejn15382-bib-0011] Engel, A. G. (2018). Congenital myasthenic syndromes in 2018. Current Neurology and Neuroscience Reports, 18, 46. 10.1007/s11910-018-0852-4 29892917

[ejn15382-bib-0012] Fulpius, B. W., Fontana, A., & Cuénoud, S. (1977). Central nervous system involvement in experimental autoimmune myasthenia gravis. The Lancet, 310, 350–351. 10.1016/S0140-6736(77)91505-7 69952

[ejn15382-bib-0013] Garcia‐Osta, A., Tsokas, P., Pollonini, G., Landau, E. M., Blitzer, R., & Alberini, C. M. (2006). MuSK expressed in the brain mediates cholinergic responses, synaptic plasticity, and memory formation. The Journal of Neuroscience, 26, 7919–7932. 10.1523/JNEUROSCI.1674-06.2006 16870737 PMC6674217

[ejn15382-bib-0014] Gautam, M., Noakes, P. G., Moscoso, L., Rupp, F., Scheller, R. H., Merlie, J. P., & Sanes, J. R. (1996). Defective neuromuscular synaptogenesis in agrin‐deficient mutant mice. Cell, 85, 525–535. 10.1016/S0092-8674(00)81253-2 8653788

[ejn15382-bib-0015] Gilhus, N. E., & Verschuuren, J. J. (2015). Myasthenia gravis: Subgroup classification and therapeutic strategies. The Lancet Neurology, 14, 1023–1036. 10.1016/S1474-4422(15)00145-3 26376969

[ejn15382-bib-0016] Gomez, A. M., Froemke, R. C., & Burden, S. J. (2014). Synaptic plasticity and cognitive function are disrupted in the absence of Lrp4. eLife, 3, e04287. 10.7554/elife.04287 25407677 PMC4270049

[ejn15382-bib-0017] Hawrylycz, M. J., Lein, E. S., Guillozet‐Bongaarts, A. L., Shen, E. H., Ng, L., Miller, J. A., Van De Lagemaat, L. N., Smith, K. A., Ebbert, A., Riley, Z. L., Abajian, C., Beckmann, C. F., Bernard, A., Bertagnolli, D., Boe, A. F., Cartagena, P. M., Chakravarty, M. M., Chapin, M., Chong, J., … Jones, A. R. (2012). An anatomically comprehensive atlas of the adult human brain transcriptome. Nature, 489, 391–399. 10.1038/nature11405 22996553 PMC4243026

[ejn15382-bib-0018] Hettwer, S., Lin, S., Kucsera, S., Haubitz, M., Oliveri, F., Fariello, R. G., Ruegg, M. A., & Vrijbloed, J. W. (2014). Injection of a soluble fragment of neural agrin (NT‐1654) considerably improves the muscle pathology caused by the disassembly of the neuromuscular junction. PLoS ONE, 9, e88739. 10.1371/journal.pone.0088739 24520420 PMC3919806

[ejn15382-bib-0019] Hodge, R. D., Bakken, T. E., Miller, J. A., Smith, K. A., Barkan, E. R., Graybuck, L. T., Close, J. L., Long, B., Johansen, N., Penn, O., Yao, Z., Eggermont, J., Hollt, T., Levi, B. P., Shehata, S. I., Aevermann, B., Beller, A., Bertagnolli, D., Brouner, K., … Lein, E. S. (2019). Conserved cell types with divergent features in human versus mouse cortex. Nature, 573, 61–68. 10.1038/s41586-019-1506-7 31435019 PMC6919571

[ejn15382-bib-0020] Huisman, S. M. H., Van Lew, B., Mahfouz, A., Pezzotti, N., Höllt, T., Michielsen, L., Vilanova, A., Reinders, M. J. T., & Lelieveldt, B. P. F. (2017). BrainScope: interactive visual exploration of the spatial and temporal human brain transcriptome. Nucleic Acids Research, 45(10), e83. 10.1093/nar/gkx046 28132031 PMC5449549

[ejn15382-bib-0021] Jung, H., Yoon, B. C., & Holt, C. E. (2012). Axonal mRNA localization and local protein synthesis in nervous system assembly, maintenance and repair. Nature Reviews Neuroscience, 13, 308–324. 10.1038/nrn3210 22498899 PMC3682205

[ejn15382-bib-0022] Karakatsani, A., Marichal, N., Urban, S., Kalamakis, G., Ghanem, A., Schick, A., Zhang, Y., Conzelmann, K.‐K., Rüegg, M. A., Berninger, B., Ruiz De Almodovar, C., Gascón, S., & Kröger, S. (2017). Neuronal LRP4 regulates synapse formation in the developing CNS. Development, 144, 4604–4615. 10.1242/dev.150110 29061639

[ejn15382-bib-0023] Keesey, J. C., Tourtellotte, W. W., Herrmann, C., Andrews, J. M., & Lindstrom, J. (1978). Acetylcholine‐receptor antibody in cerebrospinal fluid. The Lancet, 311, 777. 10.1016/s0140-6736(78)90899-1 76784

[ejn15382-bib-0024] Ksiazek, I., Burkhardt, C., Lin, S., Seddik, R., Maj, M., Bezakova, G., Jucker, M., Arber, S., Caroni, P., Sanes, J. R., Bettler, B., & Ruegg, M. A. (2007). Synapse loss in cortex of agrin‐deficient mice after genetic rescue of perinatal death. Journal of Neuroscience, 27, 7183–7195. 10.1523/JNEUROSCI.1609-07.2007 17611272 PMC6794585

[ejn15382-bib-0025] Kumar, P., Ferns, M. J., & Meizel, S. (2006). Identification of agrinSN isoform and muscle‐specific receptor tyrosine kinase in sperm. BBRC, 342, 522–528. 10.1016/j.bbrc.2006.01.161 16487930

[ejn15382-bib-0026] Kumar, P., & Meizel, S. (2005). Nicotinic acetylcholine receptor subunits and associated proteins in human sperm. Journal of Biological Chemistry, 280, 25928–25935. 10.1074/jbc.M502435200 15894803

[ejn15382-bib-0027] Lefvert, A. K., & Pirskanen, R. (1977). Acetylcholine‐receptor antibodies in cerebrospinal fluids of patients with myasthenia gravis. The Lancet, 310, 351–352. 10.1016/S0140-6736(77)91506-9 69953

[ejn15382-bib-0028] Mao, Z., Yin, J., Lu, Z., & Hu, X. (2015). Association between myasthenia gravis and cognitive function: A systematic review and meta‐analysis. Annals of Indian Academy of Neurology, 18, 131–137. 10.4103/0972-2327.156560 26019407 PMC4445185

[ejn15382-bib-0029] McMahan, U. J., Horton, S. E., Werle, M. J., Honig, L. S., Kröger, S., Ruegg, M. A., & Escher, G. (1992). Agrin isoforms and their role in synaptogenesis. Current Opinion in Cell Biology, 4, 869–874. 10.1016/0955-0674(92)90113-Q 1329871

[ejn15382-bib-0030] Miller, J. A., Ding, S.‐L., Sunkin, S. M., Smith, K. A., Ng, L., Szafer, A., Ebbert, A., Riley, Z. L., Royall, J. J., Aiona, K., Arnold, J. M., Bennet, C., Bertagnolli, D., Brouner, K., Butler, S., Caldejon, S., Carey, A., Cuhaciyan, C., Dalley, R. A., … Lein, E. S. (2014). Transcriptional landscape of the prenatal human brain. Nature, 508, 199–206. 10.1038/nature13185 24695229 PMC4105188

[ejn15382-bib-0031] Miyoshi, S., Tezuka, T., Arimura, S., Tomono, T., Okada, T., & Yamanashi, Y. (2017). DOK 7 gene therapy enhances motor activity and life span in ALS model mice. EMBO Molecular Medicine, 9, 880–889. 10.15252/emmm.201607298 28490573 PMC5494517

[ejn15382-bib-0032] Müller, K. M. I., Taskinen, E., Lefvert, A. K., Pirskanen, R., & Iivanainen, M. (1987). Immunoactivation in the central nervous system in myasthenia gravis. Journal of the Neurological Sciences, 80, 13–23. 10.1016/0022-510X(87)90217-6 3612179

[ejn15382-bib-0033] Ohno, K., Ohkawara, B., & Ito, M. (2017). Agrin‐LRP4‐MuSK signaling as a therapeutic target for myasthenia gravis and other neuromuscular disorders. Expert Opinion on Therapeutic Targets, 21, 949–958. 10.1080/14728222.2017.1369960 28825343

[ejn15382-bib-0034] Okada, K., Inoue, A., Okada, M., Murata, Y., Kakuta, S., Jigami, T., Kubo, S., Shiraishi, H., Eguchi, K., Motomura, M., Akiyama, T., Iwakura, Y., Higuchi, O., & Yamanashi, Y. (2006). The muscle protein Dok‐7 is essential for neuromuscular synaptogenesis. Science, 312, 1802–1805. 10.1126/science.1127142 16794080

[ejn15382-bib-0035] Paul, R. H., Cohen, R. A., Gilchrist, J. M., Aloia, M. S., & Goldstein, J. M. (2000). Cognitive dysfunction in individuals with myasthenia gravis. Journal of the Neurological Sciences, 179, 59–64. 10.1016/S0022-510X(00)00367-1 11054486

[ejn15382-bib-0036] Ruiter, A. M., Verschuuren, J. J. G. M., & Tannemaat, M. R. (2020). Fatigue in patients with myasthenia gravis. A systematic review of the literature. Neuromuscular Disorders, 30, 631–639. 10.1016/j.nmd.2020.06.010 32718868

[ejn15382-bib-0037] Sabre, L., Evoli, A., & Punga, A. R. (2019). Cognitive dysfunction in mice with passively induced MuSK antibody seropositive myasthenia gravis. Journal of the Neurological Sciences, 399, 15–21. 10.1016/j.jns.2019.02.001 30738333

[ejn15382-bib-0038] Sengupta‐Ghosh, A., Dominguez, S. L., Xie, L., Barck, K. H., Jiang, Z., Earr, T., Imperio, J., Phu, L., Budayeva, H. G., Kirkpatrick, D. S., Cai, H., Eastham‐Anderson, J., Ngu, H., Foreman, O., Hedehus, M., Reichelt, M., Hotzel, I., Shang, Y., Carano, R. A. D., … Easton, A. (2019). Muscle specific kinase (MuSK) activation preserves neuromuscular junctions in the diaphragm but is not sufficient to provide a functional benefit in the SOD1(G93A) mouse model of ALS. Neurobiology of Disease, 124, 340–352. 10.1016/j.nbd.2018.12.002 30528255

[ejn15382-bib-0039] Sun, X.‐D., Li, L., Liu, F., Huang, Z.‐H., Bean, J. C., Jiao, H.‐F., Barik, A., Kim, S.‐M., Wu, H., Shen, C., Tian, Y., Lin, T. W., Bates, R., Sathyamurthy, A., Chen, Y.‐J., Yin, D.‐M., Xiong, L., Lin, H.‐P., Hu, J.‐X., … Mei, L. (2016). Lrp4 in astrocytes modulates glutamatergic transmission. Nature Neuroscience, 19, 1010–1018. 10.1038/nn.4326 27294513 PMC4961622

[ejn15382-bib-0040] Tian, Q. B., Suzuki, T., Yamauchi, T., Sakagami, H., Yoshimura, Y., Miyazawa, S., Nakayama, K., Saitoh, F., Zhang, J. P., Lu, Y., Kondo, H., & Endo, S. (2006). Interaction of LDL receptor‐related protein 4 (LRP4) with postsynaptic scaffold proteins via its C‐terminal PDZ domain‐binding motif, and its regulation by Ca/calmodulin‐dependent protein kinase II. The European Journal of Neuroscience, 23, 2864–2876. 10.1111/j.1460-9568.2006.04846.x 16819975

[ejn15382-bib-0041] Tong, O., Delfiner, L., & Herskovitz, S. (2018). Pain, headache, and other non‐motor symptoms in myasthenia gravis. Current Pain and Headache Reports, 22, 39. 10.1007/s11916-018-0687-3 29725917

[ejn15382-bib-0042] Weatherbee, S. D., Anderson, K. V., & Niswander, L. A. (2006). LDL‐receptor‐related protein 4 is crucial for formation of the neuromuscular junction. Development, 133, 4993–5000. 10.1242/dev.02696 17119023

[ejn15382-bib-0043] Whiting, P., Cooper, J., & Lindstrom, J. (1987). Antibodies in sera from patients with myasthenia gravis do not bind to nicotinic acetylcholine receptors from human brain. Journal of Neuroimmunology, 16, 205–213. 10.1016/0165-5728(87)90075-0 3624454

[ejn15382-bib-0044] Yao, Z., Nguyen, T. N., van Velthoven, C. T. J., Goldy, J., Sedeno‐Cortes, A. E., Baftizadeh, F., Bertagnolli, D., Casper, T., Crichton, K., Ding, S.‐L., Fong, O., Garren, E., Glandon, A., Gray, J., Graybuck, L. T., Hirschstein, D., Kroll, M., Lathia, K., Levi, B., … Zeng, H. (2021). A taxonomy of transcriptomic cell types across the isocortex and hippocampal formation. Cell, 184(12), 3083–3085. 10.1016/j.cell.2021.04.021 34004146 PMC8195859

[ejn15382-bib-0045] Zhang, H., Sathyamurthy, A., Liu, F., Li, L., Zhang, L., Dong, Z., Cui, W., Sun, X., Zhao, K., Wang, H., Ho, H.‐Y. H., Xiong, W.‐C., & Mei, L. (2019). Agrin‐Lrp4‐Ror2 signaling regulates adult hippocampal neurogenesis in mice. eLife, 8, e45303. 10.7554/eLife.45303 31268420 PMC6650252

[ejn15382-bib-0046] Zimmermann, M. (2013). Neuronal AChE splice variants and their non‐hydrolytic functions: Redefining a target of AChE inhibitors? British Journal of Pharmacology, 170, 953–967. 10.1111/bph.12359 23991627 PMC3949645

[ejn15382-bib-0047] Zisimopoulou, P., Evangelakou, P., Tzartos, J., Lazaridis, K., Zouvelou, V., Mantegazza, R., Antozzi, C., Andreetta, F., Evoli, A., Deymeer, F., Saruhan‐Direskeneli, G., Durmus, H., Brenner, T., Vaknin, A., Berrih‐Aknin, S., Frenkian Cuvelier, M., Stojkovic, T., Debaets, M., Losen, M., … Tzartos, S. J. (2014). A comprehensive analysis of the epidemiology and clinical characteristics of anti‐LRP4 in myasthenia gravis. Journal of Autoimmunity, 52, 139–145. 10.1016/j.jaut.2013.12.004 24373505

