# Supplementary material for: Timing and localization of myasthenia gravis‐related gene expression
Source: Eur J Neurosci. 2021 Jul 20;54(4):5574–85. doi: 10.1111/ejn.15382 (PMC8457065; doi:10.1111/ejn.15382)
Supplement: Supplementary file 1 — Table S1. Genes and probes used for MG‐related genes from the AHBA. * used for analysis, unless otherwise specified. # detects the AGRN‐208 isoform able to induce AChR clustering at the NMJ. Figure S1. Expression of ACHE isoforms (A) and AGRN isoforms (B) extracted from the GTEx database. Expression of synapse‐specific ACHE‐207 isoform (red) is limited to brain areas and skeletal muscle. Expression of NMJ‐specific AGRN‐208 isoform (red) is limited to brain areas. Grey isoforms are non‐protein coding according to Ensembl (GRCh38). * isoforms identified in earlier assemblies of the human genome. Figure S2. Hierarchical clustering of gene expression in the GTEx database using all isoforms of ACHE and AGRN. Figure S3. Heatmaps from Allen human brain atlas. Relative expression of different agrin probes across distinct brain areas (A). # probe that selectively detects NMJ‐relevant isoform of agrin. * probe for AGRN used in the BrainScope tool detecting all AGRN isoforms. Hierarchical clustering using probe for AGRN that does not discriminate between isoforms (*) (B). Figure S4. Gene expression of MG‐related genes during human development do not show the same pattern across development. CHRNA1 expression is increased across development restricted to the hippocampus. DOK7 shows a pattern of increasing expression across development, with highest expression in the cerebellum and thalamus. MUSK is lowly expressed during prenatal development in the hippocampus, amygdala and cerebellum. RAPSN shows very limited expression across development, restricted to some subcortical regions of the brain. Visualized using the BrainSpan portal. [file EJN-54-5574-s001.pdf]

Supplemental material

Supplemental Table 1. Genes and probes used for MG-related genes from the AHBA. * used for analysis, unless otherwise specified. # detects the AGRN-208 isoform able to induce AChR clustering at the NMJ.

**Gene names**	**Allen Brain Atlas probes**
ACHE	A_24_P60845*
AGRN	A_23_P343411*<br>A_24_P24819<br>A_24_P358462#<br>CUST_72_PI416408490
CHRNA1	A_23_P90888*<br>CUST_14430_PI416261804
COLQ	A_23_P212126*<br>A_24_P26160
DOK7	A_23_P39885*<br>CUST_10065_PI416261804
LRP4	A_24_P403561*<br>CUST_9952_PI416261804
MUSK	A_23_P71649*<br>A_24_P129536
RAPSN	A_23_P86801<br>CUST_899_PI416261804*

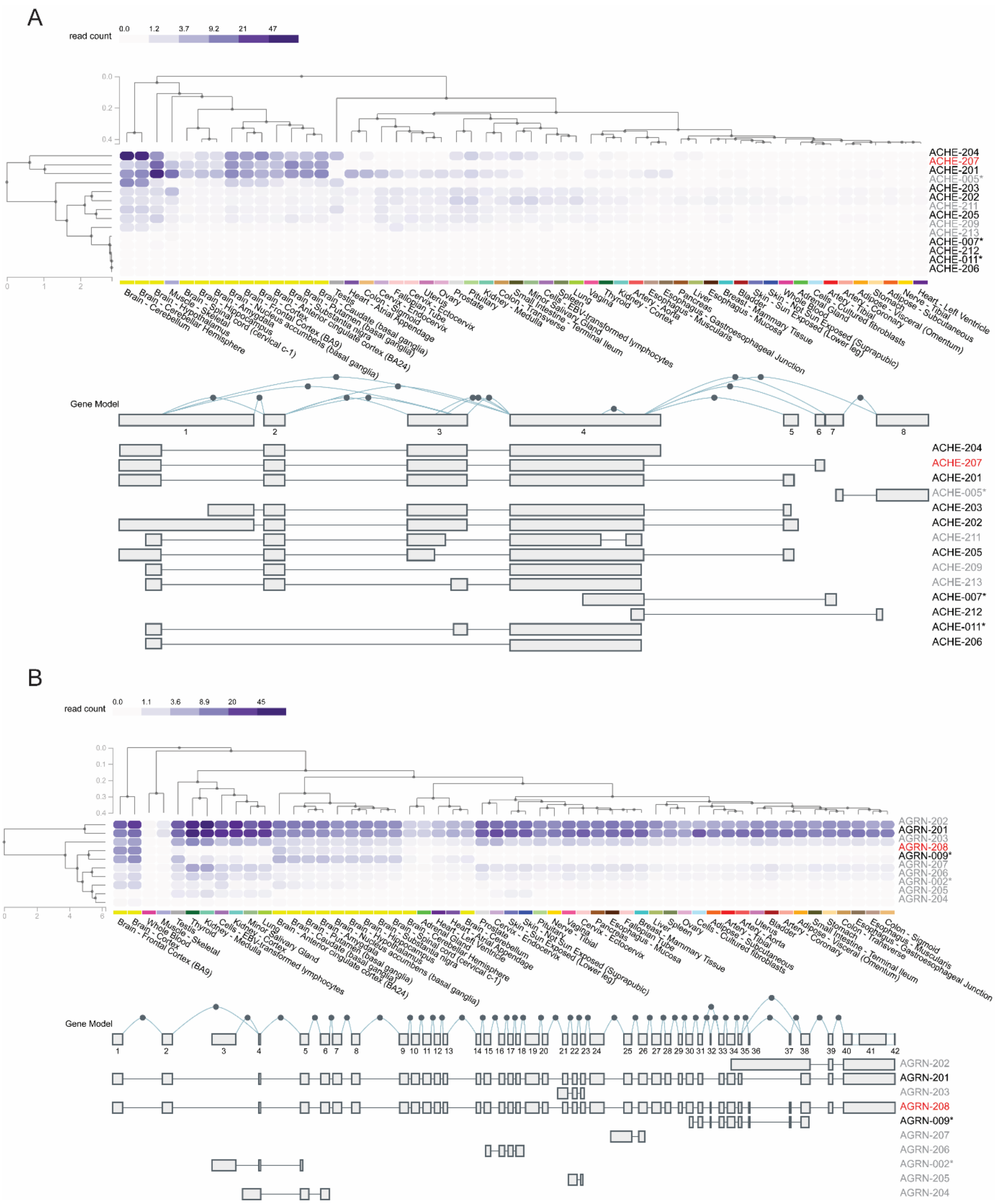

Supplemental Fig. 1. Expression of *ACHE* isoforms (A) and *AGRN* isoforms (B) extracted from the GTEx database. Expression of synapse-specific *ACHE-207* isoform (red) is limited to brain areas and skeletal muscle. Expression of NMJ-specific *AGRN-208* isoform (red) is limited to brain areas. Grey isoforms are non-protein coding according to Ensembl (GRCh38). * isoforms identified in earlier assemblies of the human genome.

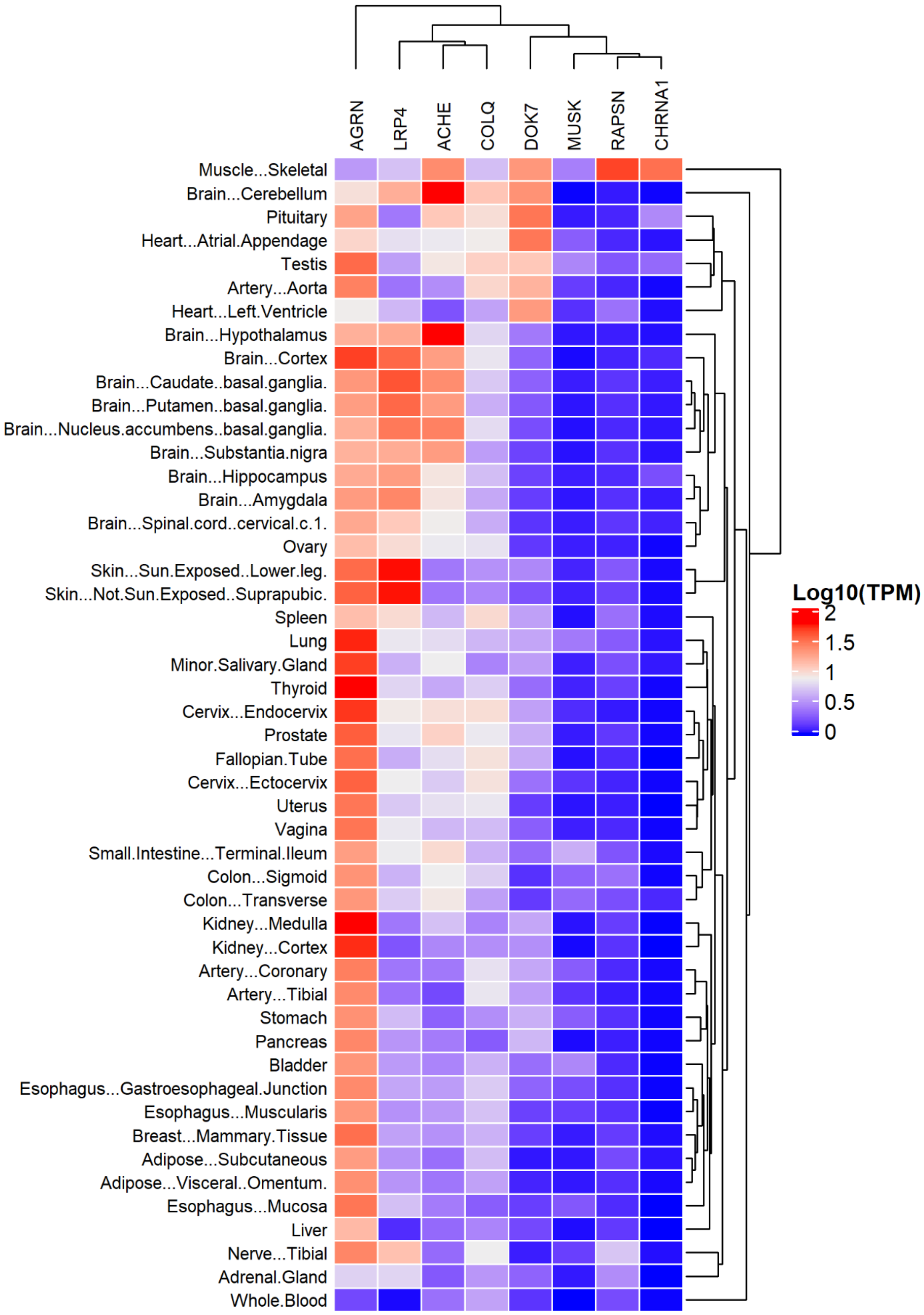

Supplemental Fig. 2. Hierarchical clustering of gene expression in the GTEx database using all isoforms of *ACHE* and *AGRN*.

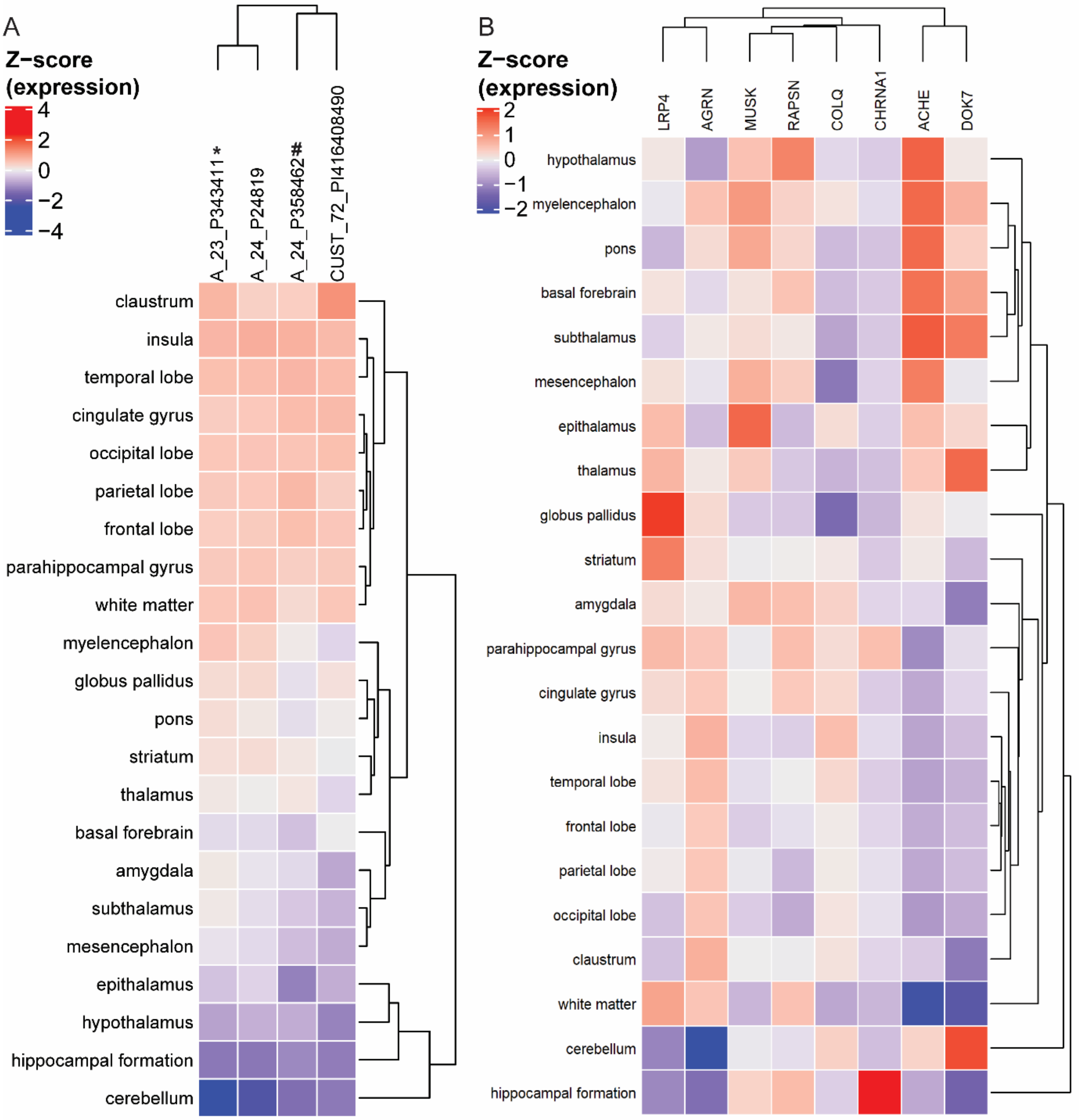

Supplemental Fig. 3. Heatmaps from Allen human brain atlas. Relative expression of different agrin probes across distinct brain areas (A). # probe that selectively detects NMJ-relevant isoform of agrin. * probe for AGRN used in the BrainScope tool detecting all AGRN isoforms. Hierarchical clustering using probe for AGRN that does not discriminate between isoforms (*) (B).

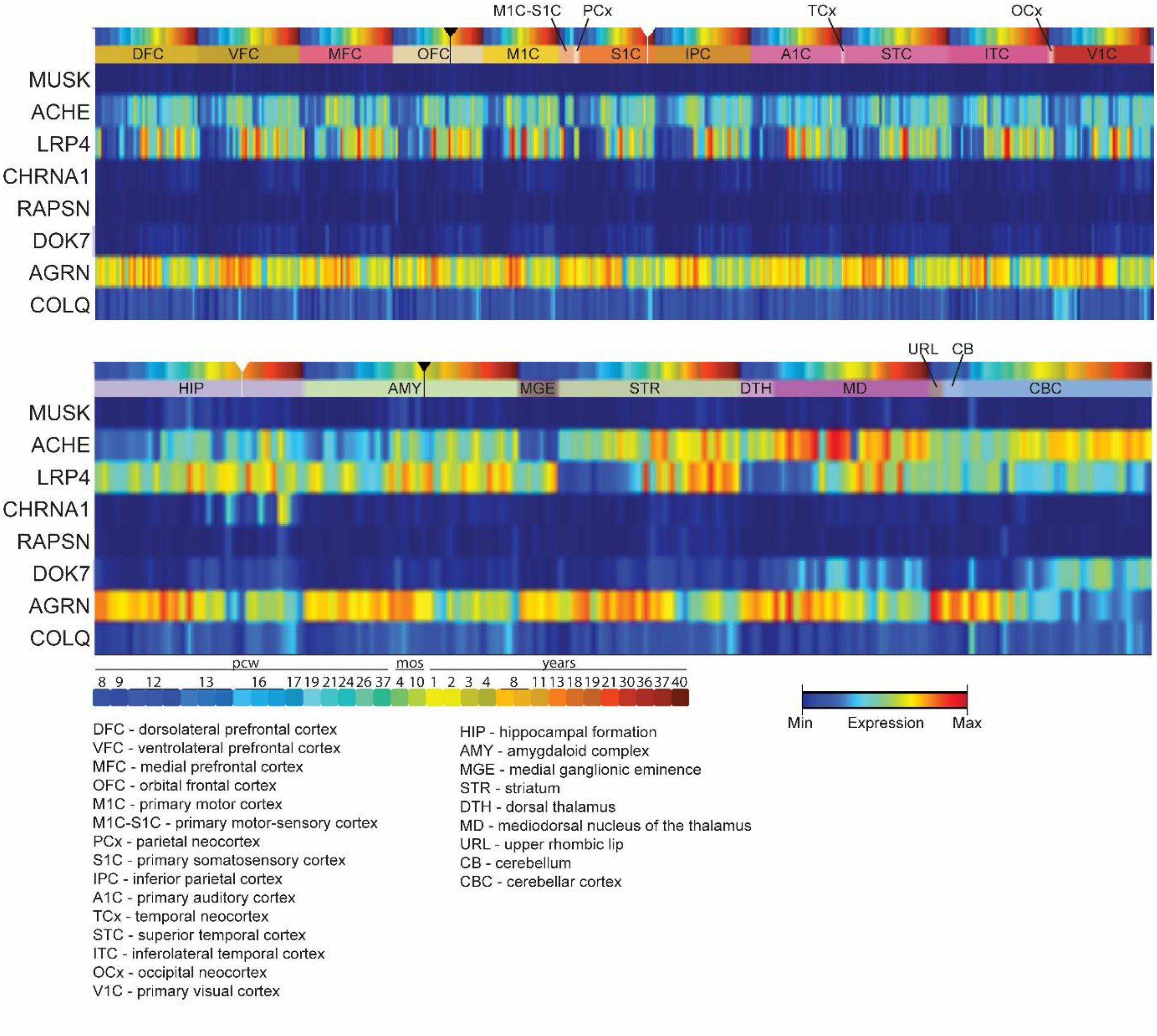

Supplemental Fig. 4. Gene expression of MG-related genes during human development do not show the same pattern across development. *CHRNA1* expression is increased across development restricted to the hippocampus. *DOK7* shows a pattern of increasing expression across development, with highest expression in the cerebellum and thalamus. *MUSK* is lowly expressed during prenatal development in the hippocampus, amygdala and cerebellum. *RAPSN* shows very limited expression across development, restricted to some subcortical regions of the brain. Visualized using the BrainSpan portal.
